# Assessment of Efficacy and Toxicity of Cyclophosphamide Chemotherapy in Canines with Malignant Mammary Tumor: A Retrospective Study

**DOI:** 10.1155/2021/5520603

**Published:** 2021-08-12

**Authors:** R. V. Suryawanshi

**Affiliations:** Assistant Professor of Surgery, Department of Veterinary Surgery and Radiology, Krantisinh Nana Patil College of Veterinary Science Shirwal, Dist-Satara-412801, Maharashtra Animal and Fishery Sciences University, Nagpur, Maharashtra, India

## Abstract

Surgical excision with chemotherapy is a commonly used treatment modality to treat canine mammary tumor (CMT), but it is unclear whether different treatment modalities may have similar efficacies and toxicities. The objective of this clinical study was to evaluate the efficacy and toxicity of cyclophosphamide chemotherapy along with surgical excision of malignant mammary tumor in canines by clinical, haemato-biochemical, radiographical, and histopathological evaluation before and after treatment. Eighteen dogs with malignant mammary tumor, reported to Teaching Veterinary Hospital, were divided into two groups consisting of nine dogs in each group. Group I (*n* = 9) dogs were treated with surgical excision of malignant mammary tumor alone, and group II (*n* = 9) was treated with surgical excision of mammary tumor with cyclophosphamide chemotherapy at 50–100 mg/m^2^ intravenously in weekly doses by three consecutive weeks. In group II, 7 dogs (78%) showed complete regression of tumor after the third dose of cyclophosphamide and showed increase in the quality and survival life and remaining two dogs showed recurrence of tumor after one year. Some dogs showed common adverse reactions such as lethargy, moderate alopecia, vomiting, anorexia, anemia, and haematuria after the third dose of chemotherapy. To conclude, surgical excision combined with cyclophosphamide chemotherapy is an effective protocol for management of malignant mammary tumor in canines with minimal toxicity and it could be possible to increase the quality and survival life of patients.

## 1. Introduction

In canines, mammary tumors are the second most frequently encountered spontaneous neoplasms following those derived from the skin [1]. It must be stated that about 41% to 53% of mammary neoplasms are malignant in nature [2, 3]. Mammary gland tumors are the most common type of tumors in unspayed females aged between 7 and 10 years with a sharp increase in incidence after five years of age, hence called onset of “cancer age” [3, 4]. Intact bitches (42 of 46) had frequent incidence of mammary tumors, and among them, 37 had abnormal reproductive life (none or few pregnancies, prevention of mating, estrus irregularities, and pseudo-pregnancies) either one or two times as a feature [5]. In small animals, mammary cancer chemotherapy is not routinely performed, except for some cases of distant metastases or tumors not amenable to surgery. Clinical research on adjuvant chemotherapy provided only a few promising results on dogs [6].

Chemotherapeutic drugs attack the cancer cells, and this emphasizes that survival rate and quality of life after chemotherapy could be prolonged in cancerous patients [7, 8]. Removal of the tumor tissue with surgical attempts is only curative for small, noninvasive, grade 1 tumors. In the treatment of large, adherent-to-the-surrounding-tissue, malignant tumors, detected to be highly metastatic by histopathologic diagnosis techniques, adjunct chemotherapy with surgical procedures is mandatory [7].

Cyclophosphamide is the most frequently used alkylating agent in veterinary medicine. Alkylating agents induce their cytotoxic effect by replacing a hydrogen atom on a biologically active molecule with an alkyl radical (R–CH2-CH3+) interfering with DNA replication and RNA transcription [6]. The efficacy of chemotherapy was judged on the basis of obvious regression of tumor growths, delayed appearance of metastases, and prolonged duration of patient's life [9, 10]. Chemotherapy always accompanied with side effects such as vomiting, diarrhea, anorexia, lethargy, fever, alopecia, anemia, and neutropenia [11, 12]. The objectives of the present study were to investigate the efficacy and side effects of cyclophosphamide chemotherapy along with surgical excision of malignant mammary tumor in canines and its follow-up strategy.

## 2. Materials and Methods

### 2.1. Selection of Animals

**Figure 1:**
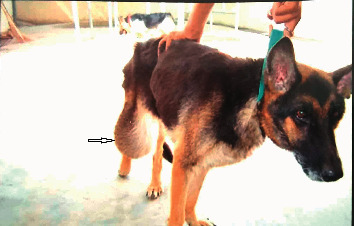
Inguinal mammary tumor (hanging in the inguinal region) in a 11-year-old German shepherd dog.

**Figure 2:**
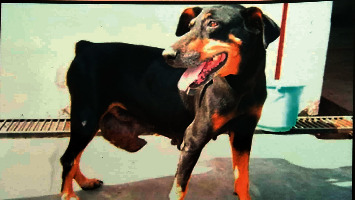
A one-year-old female Doberman having inguinal mammary tumor with cauliflower appearance.

The present clinical study was carried out on 18 female dogs of variable age groups (2–14 years) and body weights (12–38 kg), belonging to various breeds like German Shepherd (5; Figure 1), Doberman (2), Pomeranian (2), Pomeranian cross (2), Lassa Apso (2), Labrador (1), nondescript (2), and Dachshund (2). All dogs were presented to the Teaching Veterinary Clinical Campus with a history of variable swelling or growth on mammary glands, partial-to-complete anorexia, generalized dullness, and weakness. Distribution mammary tumor in all dogs includes the following: the inguinal pair was highly affected with tumor (7 cases; Figure 2) followed by caudal abdominal (4 cases); cranial abdominal and cranial thoracic glands (3 cases each); and caudal thoracic gland (1 case). Data related to duration of illness, weight of the tumor, neutering status of the dog, gross observation of the tumor, and clinical symptoms were recorded during initial screening. All eighteen dogs were subjected into two groups comprising nine animals in each group.

### 2.2. Radiographic Examination

Of 18, seven dogs with malignant mammary tumor accompanied with respiratory distress underwent routine lateral thoracic radiographic examination. The efficacy of chemotherapy was judged on the basis of severity of lung metastasis, pattern of nodular intensity, and regression of tumor before and after cyclophosphamide chemotherapy.

### 2.3. Haemato-Biochemical Study

About 4-5 mL of blood was collected and mixed with EDTA and serum vial for estimation of total erythrocyte count, total leucocyte count, haemoglobin, packed cell volume, platelet count, serum glutamic pyruvate transaminase/AST, serum glutamic oxaloacetate transaminase/ALT, blood urea nitrogen, and serum creatinine at the 0^th^, 1^st^, 3^rd^, 7^th^, 14^th^, and 21^st^ days of interval to assess the haemato-biochemical alteration in group II dogs affected with mammary tumor irrespective of their histological nature.

### 2.4. Histopathological Examination of the Tumor

Tissue samples from mammary tumor were collected from all dogs via the excision biopsy method and fixed in 10% neutral buffer formalin, washed, dehydrated, and then fixed in paraffin. About 3-4*µ*m-thick sections were stained with H&E stain and examined under a light microscope as per guidelines of Sales Lapa et al. [13].

  Treatment protocol of group I: dogs with malignant mammary tumor (*n* = 9) were confirmed histopathologically as well as by thoracic radiography and were subjected to complete surgical excision of mammary tumor alone and their occurrence was recorded during investigation.
  Treatment protocol of group II: Dogs with malignant mammary tumor (*n* = 9) were confirmed histopathologically as well as by thoracic radiography and were subjected to complete surgical excision of mammary tumor coupling with three doses of cyclophosphamide chemotherapy at the dose rate of 100 mg/m^2^ along with intravenous fluid at a weekly interval of 3 weeks as per the body surface formula described in [14] and by Karayannopoulou et al. [15].

### 2.5. Response to Treatment

In group II, all dogs were evaluated on the basis of clinical symptoms, regression of tumor into the lung via thoracic radiography, recurrence of tumor at the surgical site, survival period of patients, adverse effects of chemotherapy, and sequelae of treatment or surgical intervention, if any were recorded during the follow-up period.

### 2.6. Statistical Analysis

The clinical data obtained from different parameters in both the groups during the present investigation were subjected to Student's *t*-test for comparison of treatment efficacies [16].

## 3. Results and Discussion

### 3.1. Incidence of Mammary Tumor

**Figure 3:**
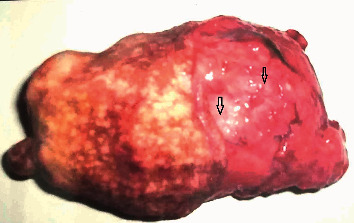
Gross appearance of resected mammary tumor showing ulcerative and hemorrhagic spots on surface weighing about 850 gm.

Incidence of mammary tumor in the present study was more common in older age (8–11 years) and unspayed female dogs. The inguinal pair of mammary gland (38.90%) was more commonly affected with tumor followed by cranial abdominal (16.67%), cranial thoracic (16.67%), caudal abdominal (22.22%), and caudal thoracic glands (5.55%), as depicted in Figures 1 and 2. The shape of the mammary tumor was round to cauliflower with hard-to-elastic consistency, attached to the abdominal and inguinal region in 11 dogs. In 4 cases, the growth was painful with rear leg edematous swelling and enlargement of the regional lymph node. One German Shepherd dog had a round-to-ovoid growth and well-circumscribed, hard lobulated, and encapsulated structure with ulcerative and hemorrhagic spots weighing about 850 gm (Figure 3). Of 18, 12 cancerous growths showed gross lesions like hemorrhagic spots, ulceration, and open wound with necrotic foci.

In the present study, incidence of mammary tumor was observed in the unspayed age group of 8–10 years corroborating with findings of Broady et al. [2], Fidler and Broday [17], and Bolzisar et al. [18], whereas Karayannopoulou et al. [5] reported that maximum incidence of mammary gland tumors was observed between 8 and 13 years of age. In the present study, inguinal glands were most commonly affected with tumor followed by caudal abdominal glands which could be due to the greater volume of glandular tissue to react to any carcinogenic irritant [1]. In the current investigation, German Shepherd, Pomeranian, nondescript, and Doberman breeds of dogs were commonly affected. However, there is little correlation between prevalence of mammary tumor and breeds of dogs in a population [19, 20]. Early ovariohysterectomy plays an important role in prevention of the occurrence of mammary tumor and increase in the survival rate of female dogs [5].

### 3.2. Thoracic Radiography

**Figure 4:**
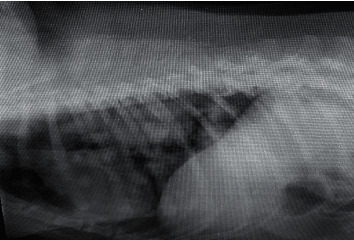
Lateral radiograph in a female Labrador revealed the presence of multiple nodular densities on the lung surface indicative of lung metastasis.

**Figure 5:**
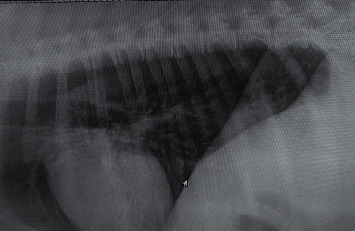
Lateral radiograph of a female Labrador showed complete regression of lung metastasis after the third dose of cyclophosphamide therapy.

Radiography of the lungs showed identical changes in seven dogs having respiratory distress characterized by multiple sharp outlined nodular densities with variable diameters (Figure 4) before surgico-chemotherapeutic treatment. Post-chemotherapeutic treatment radiographs of the same dogs revealed significant reduction of lung field density and decrease in the size and intensity of nodules in the lungs, indicating clear reduction of lung metastasis and appearance of normal lung field (Figure 5) in group II. Thoracic radiographs of seven dogs in the present study showed presence of nodules with variable densities, indicating tumor metastasis in alveoli of the lungs. Detection of lung metastasis via thoracic radiography is very sensitive and accurate and of prognostic value, as described by Tiemessen [21]. The lung is the most common site for distant metastasis in dogs with malignant mammary tumor [7]. In the present study, lung metastases were recorded and their regression was followed up prior to and at the end of chemotherapy according to Hershey et al. [22].

### 3.3. Haemato-Biochemical Analysis

The total erythrocyte count was decreased in group II on the 3^rd^ day (5.26 ± 0.56) as compared to the 0^th^ day (6.38 ± 0.12), and again, it was restored on the 21^st^ day (7.38 ± 0.09) revealing nonsignificant changes. Leucopenia (13.8 ± 0.45) was observed after the second dose of cyclophosphamide in few dogs. The average level of haemoglobin (13.8 ± 0.25 gm%–13.1 ± 0.07 gm %), packed cell volume (35.7 ± 0.30%), and differential leucocyte count was fluctuating and nonsignificant throughout the present investigation. In group II, the average mean value of platelet count on the 0^th^ day was 2.66 ± 0.01 and it was gradually reduced on the 1^st^ (1.55 ± 0.06), 3^rd^ (1.48 ± 0.07), 7^th^ (1.57 ± 0.06), 14^th^ (1.51 ± 0.08), and 21^st^ day (1.56 ± 0.10) indicating severe thrombocytopenia during the course of cyclophosphamide chemotherapy (Table 1). Blood profile revealed nonsignificant fluctuation of haemoglobin, and packed cell volume was recorded in collaboration with Benzamin [23] who also recorded nonsignificant decrease in Hb and PCV values. However, significant decrease in TLC was observed after the third dose of cyclophosphamide chemotherapy. Todorova et al. [14] reported significant decrease in leucocyte count after every cycle of doxorubicin and cyclophosphamide therapy when compared to the values before surgery.

The mean serum level of AST values was in the range between 37.4 ± 0.60 and 37.5 ± 0.42, whereas ALT values were in the range of 38.5 ± 0.11 to 47.3 ± 0.10 showing significant changes. The blood urea nitrogen level was significantly increased in group II dogs on the 21^st^ day (25.9 ± 0.68) as compared to the 0^th^ day (20.4 ± 0.58). The serum creatinine level also elevated (1.56 ± 0.02) after the third dose of cyclophosphamide in all dogs as compared to the 0^th^ day (1.11 ± 0.04) (Table 2). There was a significant increase in serum values of BUN (20.4 ± 0.58 to 25.9 ± 0.66) and creatinine values (1.1 ± 0.04 to 1.6 ± 0.03) in animals of group II after the third dose of cyclophosphamide as compared to the 0^th^ day (pretreatment). Riley and Riley [24] reported progressive increase in BUN throughout the period of chemotherapy with vincristine, cyclophosphamide, and methotrexate.

The mean serum values of AST were in the normal range before and after surgical treatment in both the groups. In animals of group II, there was a significant increase in the ALT serum values (38.5 ± 0.11 to 47.3 ± 0.10) after the 2^nd^ and 3^rd^ dose of cyclophosphamide which could be due to detoxification process of cyclophosphamide in the liver [14, 23]. In contrast, Palta [25] reported nonsignificant changes in AST and ALT values after vincristine chemotherapy in mammary tumor.

### 3.4. Treatment Response

Clinical findings such as body temperature, heart rate, and respiratory rate did not show considerable changes during the present investigation (Table 2) in both the groups of dogs. In group I, five female dogs including Pomeranian, German Shepherd, and nondescript aged between 7 and 12 years did not show recurrence of tumor after one-year follow-up. Two dogs showed moderate-to-severe respiratory distress probably due to lung metastasis, and they were euthanized as per the owner's request, and the remaining two dogs underwent second surgical excision of mammary tumor. However, in group II, six dogs showed normal appetite till the first week and after that, there was slight reduction in appetite with vomiting and retching and they were treated symptomatically. In group I, of 9, two dogs showed recurrence or progressive disease after surgical excision as compared to group II dogs.

**Figure 6:**
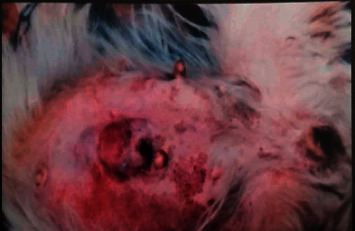
An 8-year-old Pomeranian showed mild-to-moderate skin rashes on the ventral abdominal region after the second dose of cyclophosphamide with recurrence of tumor.

**Figure 7:**
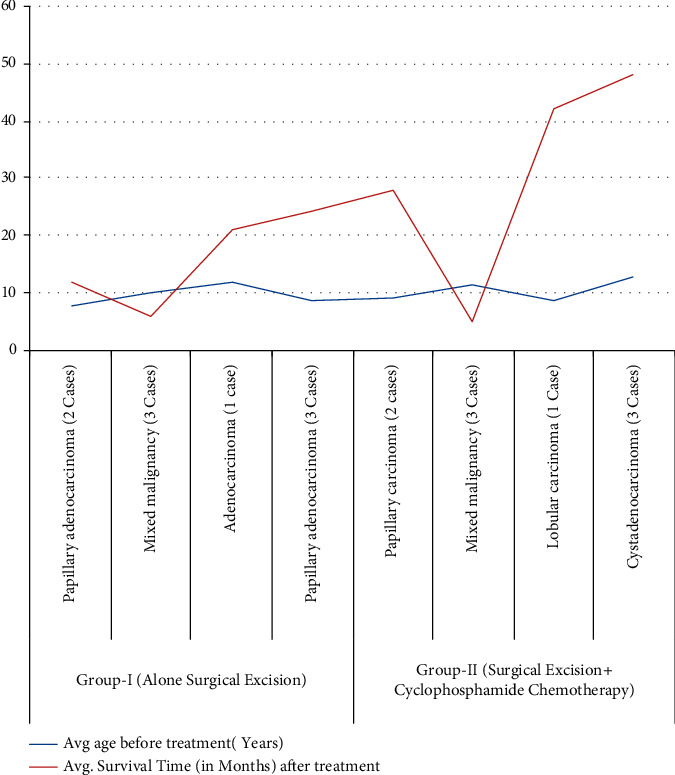
Line bar showing average survival time in group I and group II in the present clinical study.

Following chemotherapy in group II, we evaluated (grades 1 to 5 with clinical description of severity) the cyclophosphamide therapy in dogs with malignant mammary tumor as depicted in Table 3. Of 9, 4 dogs showed intermittent nausea, skin rashes, vomiting, diarrhea, and mild-to-moderate alopecia during the course of chemotherapy. Few dogs showed gastrointestinal disturbances during the chemotherapeutic course, and they were treated with Ringer's lactate, metronidazole, antacids, and sucralfate suspension for weeks and recovered uneventfully. The remaining three dogs showed severe haematuria after the second dose of cyclophosphamide and were treated symptomatically. One dog showed moderate recurrence of tumor after chemotherapy with severe skin rashes (Figure 6). Results indicated that dogs treated with surgical excision alone showed higher incidence of recurrence of tumor than dogs treated with surgical excision along with cyclophosphamide chemotherapy in the present study, suggesting that the survival time (Figure 7) was higher in group II in respect to age, signalment, and histopathological grade and their quality of life was also improved as per the owner's information. Nonsignificant changes were recorded in physiological parameters in animals of group II during cyclophosphamide chemotherapy [14]. In group I, five dogs showed complete recovery after mammectomy and there was recurrence of tumor in two nondescript dogs probably due to metastasis. Gultiken and Vural [26] and Chang et al. [27] reported that tumors which are developed in the center of the mammary lobe can be removed via mammectomy and longevity of life after the operation will be increased if all the tumor tissues were removed. In animals of group II, general condition of 6 dogs was good and stable throughout treatment with nonsignificant changes in digestive parameters like appetite, vomiting, lethargy, and diarrhea that could be due to mild ulceration of gastric mucosa [11, 28, 29]. Skin reaction and alopecia were recorded in one dog after therapy [30]. Of 9, one dog showed haematuria after the second dose of cyclophosphamide which could be due to nephrotoxicity of the cyclophosphamide drug [31].

In the present study, removal of malignant mammary tumor via surgical excision along with cyclophosphamide showed favorable results and was found to be an efficient modality [12, 14, 32–38]. The follow-up strategy of animals in group II showed clear regression of tumor in the lungs, no recurrence of growth, and increased survival period of the patient showing worthiness towards the management of malignant tumor in canines [9, 10, 29].

### 3.5. Histopathological Examination

**Figure 8:**
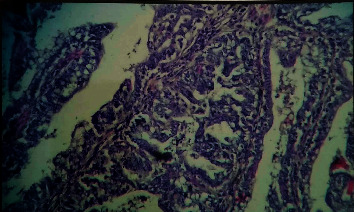
Histopathological appearance of mammary tumor showed multiple ductlike structures filled with coalescing multibranched papillae.

**Figure 9:**
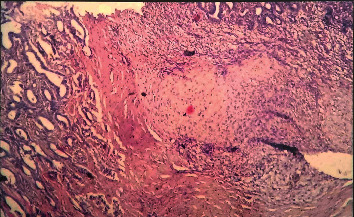
Solid carcinoma of the mammary gland in a 9-year-old Doberman female (H&E, x100).

Of 18, 13 tumors (72.22%) were malignant in nature including papillary carcinoma, mixed malignancy tumor, lobular carcinoma, and cystadenocarcinoma and the remaining five tumors were of benign type. Histopathologically, five mammary tissues showed multiple ductlike structures filled with coalescing multibranched papillae (Figure 8); four dogs showed mixed malignant tumor characterized by cartilaginous or osseous components and involvement of partial or complete gland; and the remaining three dogs had lobular carcinoma composed of undifferentiated, often small and hyperchromatic, cells with lobular and ductile alveoli due to proliferating tumor cells (Figure 9). Dore et al. [39] reported that 50% of mammary tumors are malignant. However, in the present study, seven dogs showed lung metastasis in accordance with Benzamin et al. [40] who reported that 36% of malignant mammary tumors may metastasize into the lungs. Thus, dogs that underwent surgical excision of mammary tumor along with cyclophosphamide chemotherapy showed reduction in the recurrence rate of tumor because of action of cyclophosphamide on the remnant part of cancerous tissue which was left during the surgical procedure.

## 4. Conclusions

The present clinical study demonstrates that minimally invasive systemic therapy performed as adjuvant to surgery with cyclophosphamide chemotherapy is a most effective protocol for management of malignant mammary tumor in dogs, but this adjunctive therapy is always accompanied with minimal toxicity which can be treated symptomatically in day-to-day canine practices which improve the quality of patient life.

## Figures and Tables

**Table 1:** Mean ± SE values of haemato-biochemical parameters in dogs with malignant mammary tumor prior to and after cyclophosphamide chemotherapy in group II dogs.

Haemato-biochemical parameters	0^th^ day (before treatment)	1^st^ day (I^st^ dose of chemotherapy)	3^rd^ day	7^th^ day (II^nd^ dose of chemotherapy)	14^th^ day (III^rd^ dose of chemotherapy)	21^st^ day
TEC (10^6^/cumm)	6.38 ± 0.12	5.82 ± 0.11	5.26 ± 0.56	6.23 ± 0.22	7.05 ± 0.15	7.38 ± 0.09
Hb (gm %)	13.8 ± 0.25	11.6 ± 0.36	11.9 ± 0.33	11.8 ± 0.20	12.6 ± 0.18	13.1 ± 0.07
PCV (%)	37.2 ± 0.55	35.7 ± 0.30	36.1 ± 0.47	35.1 ± 0.48	35.9 ± 0.33	36.6 ± 0.36
TLC (thousand/cumm)	15.7 ± 0.44	15.9 ± 0.31	13.8 ± 0.45	16.8 ± 0.45	16.3 ± 0.53	14.6 ± 0.27
Platelet (lakh/cumm)	2.66 ± 0.1	1.55 ± 0.06	1.48 ± 0.07	1.57 ± 0.06	1.51 ± 0.08	1.56 ± 0.10
AST (IU/L)	37.4 ± 0.60	38.3 ± 0.34	38.1 ± 0.26	38.6 ± 0.34	38.1 ± 0.32	37.5 ± 0.42
ALT (IU/L)	38.5 ± 0.11	47.4 ± 0.52	47.1 ± 0.28	47.6 ± 0.19	47.5 ± 0.29	47.3 ± 0.10
BUN (mg/dl)	20.4 ± 0.58	21.9 ± 0.49	22.8 ± 0.39	24.9 ± 0.38	27.1 ± 0.13	25.9 ± 0.68
Serum creatinine (mg/dl)	1.11 ± 0.04	1.41 ± 0.06	1.56 ± 0.02	1.65 ± 0.03	1.47 ± 0.05	1.56 ± 0.02

**Table 2:** Mean ± SE values of clinical/physiological parameters in dogs with malignant mammary tumor prior treatment in both the groups at periodical intervals.

Treatment group	Clinical parameter	0^th^ (day)	1^st^ (day)	3^rd^ (day)	7^th^ (day)	14^th^ (day)	21^st^ (day)
I	Body temperature (°C)	38.12 ± 1.092	37.00 ± 1.000	37.84 ± 1.014	38.12 ± 6.310	38.12 ± 4.200	37.23 ± 1.218
	Heart rate (/min)	118.00 ± 14.5	117.12 ± 10.00	119.21 ± 63.45	116.01 ± 42.10	120.11 ± 21.00	119.00 ± 32.10
	Respiratory rate (/min)	47.21 ± 15.21	50.00 ± 54.21	49.10 ± 21.01	48.21 ± 34.21	52.13 ± 10.00	49.14 ± 82.16

II	Body temperature (°C)	37.82 ± 1.082	38.14 ± 1.142	37.42 ± 2.101	39.25 ± 3.210	40.00 ± 1.092	39.56 ± 1.241
	Heart rate (/min)	120.01 ± 51.2	118.21 ± 41.22	115.63 ± 25.12	118.00 ± 10.21	117.13 ± 63.24	120.56 ± 32.10
	Respiratory rate (/min)	46.12 ± 72.16	49.24 ± 56.21	50.12 ± 32.10	52.13 ± 54.12	49.15 ± 34.23	48.24 ± 11.00

**Table 3:** Grading of postchemotherapeutic complications/toxicities in group II dogs (according to VCOG-AE criteria).

Grades	Nature of symptoms	Toxicity/symptoms	No. of dogs involved
Grade 1	Mild nature	Intermittent nausea, skin rashes, vomiting, diarrhea, mild alopecia	4
Grade 2	Moderate	Moderate alopecia, haematuria	2
Grade 3	Severe	Intermittent haematuria, persistent vomiting, severe dehydration	2
Grade 4	Life threatening	Anemic, moribund state, thrombocytopenia	1
Grade 5	Death	—	

## Data Availability

Data will be made available on request through a data access committee, institutional review board, or the author.
